# Supplementary material for: Chemically identifying single adatoms with single-bond sensitivity during oxidation reactions of borophene
Source: Nat Commun. 2022 Apr 4;13:1796. doi: 10.1038/s41467-022-29445-8 (PMC8979967; doi:10.1038/s41467-022-29445-8)
Supplement: Supplementary file 1 — Supplementary Information [file 41467_2022_29445_MOESM1_ESM.pdf]

Supplementary Information

Chemically identifying single adatoms with single-bond sensitivity during oxidation reactions of borophene

*Linfei Li[1], Jeremy F. Schultz[1], Sayantan Mahapatra[1], Zhongyi Lu[1], Xu Zhang[2], and Nan Jiang[1]**

[1]Department of Chemistry, University of Illinois Chicago, Chicago, Illinois 60607, USA.
[2]Department of Physics and Astronomy, California State University, Northridge, Northridge, California 91330, USA.
**Author to whom correspondence should be addressed: njiang@uic.edu*

Supplementary Discussion

1. Interrogation of the nature of big clusters on oxidized borophene

Although uniform oxygen adatoms were predominant on atomic-oxygen-oxidized borophene surfaces, a few bright protrusions were present near borophene edges. We can attribute them to oxygen clustering and resulting boron oxide species due to the considerable mobility of atomic oxygen on borophene[1]. In a controlled experiment, we exposed borophene surfaces to 6 L of atomic oxygen at an elevated temperature (85 °C). As seen in Supplementary Fig. 2, borophene edges are significantly oxidized with a reduced coverage of oxygen adatoms on borophene terraces. These features are in sharp contrast to those of oxidized borophene prepared at room temperature as shown in Fig. 1h. This results from the enhanced lateral mobility of atomic oxygen at elevated temperatures and the strong thermodynamic and kinetic driving forces that lead to oxygen adsorption and boron oxidation at borophene edges due to rich dangling bonds therein. Supplementary Fig. 3 presents a borophene edge decorated with clusters. We measured one of these clusters with TERS, and found two peaks as shown in red. Although the mode located at 733 cm^{-1} has not been defined, the 875 cm^{-1} peak can be assigned to the breathing mode of the three oxygen atoms within a B_2O_3 structure[2]. These results suggest that boron oxides are a minority species following the oxidation of borophene using atomic oxygen at room temperature.

2. Effect of Ag substrates on the phonons of oxygen adsorbed borophene

Despite a weak interaction between borophene sheets and Ag substrates, the electron transfer doping from Ag to borophene has been demonstrated experimentally[3], which potentially affects the vibrational properties of borophene and adsorbates on it. In our previous study, organic molecules (tetraphenyldibenzoperiflanthene, DBP) were deposited on borophene in the form of physical adsorption[4]. Due to the negligible charge transfer between DBP and borophene, the measured TERS spectra of adsorbed DBP molecules were consistent with those of pristine (gas-phase) DBP. By contrast, in the present system oxygen adatoms are covalently bound to borophene via B–O bonds. As a result, the electrons donated by the Ag substrate will readily charge the B–O bonds and thus modulate the phonon frequencies of oxygen-modified Raman modes. Consequently, phonon

simulations based on an isolated borophene without considering interactions with Ag substrates reasonably give rise to Raman frequencies with a small deviation from experimental values.

3. Additional discussion for tip-induced decomposition of BO_x clusters

Supplementary Fig. 13 presents STM images of the same area as that shown in Fig. 6 but at a larger scale. Cluster 1 is the same one as shown in Fig. 6b. In Supplementary Fig. 13b, we can see that scanning at 4.0 V resulted in the degradation of Cluster 1 into a small and low protrusion. This topographic change was not attributed to an accidental variation of tip conditions and thus an imaging artifact, because the same reaction happened subsequently to Cluster 2 under the same scanning conditions. Additionally, aside from the decomposed clusters, the STM topography before and after the reactions appears consistent without sudden changes or sharp contrast. Consequently, Supplementary Fig. 13b and Fig. 6c demonstrate tip-induced decomposition reactions of BO_x clusters.

Supplementary Figures

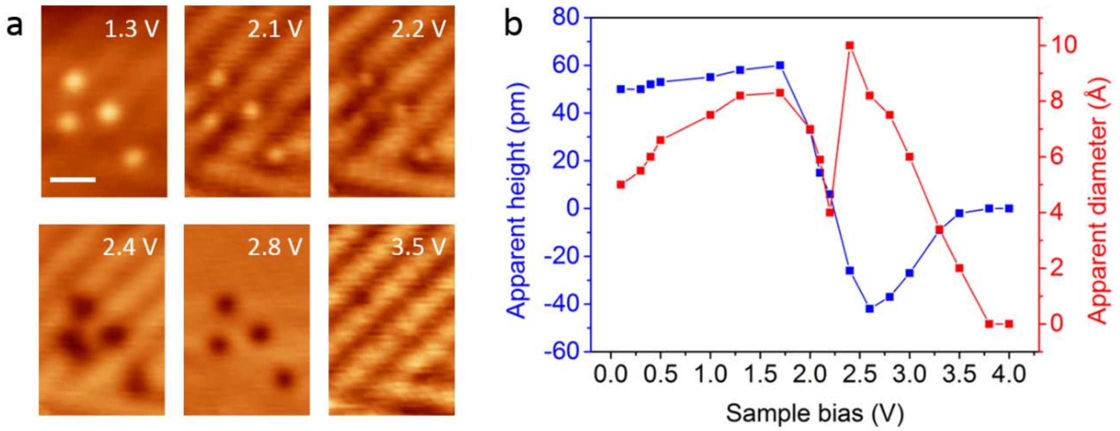

Supplementary Figure 1. Bias dependence of the topography of oxygen adatoms on borophene. a, STM images of four oxygen adatoms on borophene scanned with different sample biases as indicated and the same tunneling current of 300 pA. Scale bar: 2 nm. **b,** Plot of the bias dependence of the apparent height and diameter of oxygen adatoms.

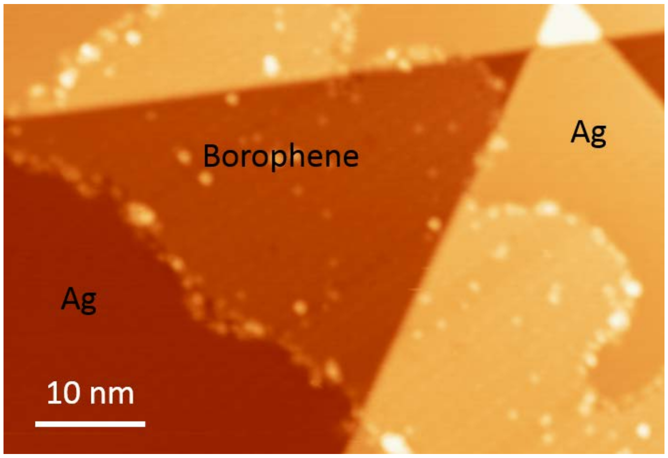

Supplementary Figure 2. STM image of a borophene island after exposure to 6 L of atomic oxygen at 85 °C. Scanning conditions: 1.0 V, 100 pA.

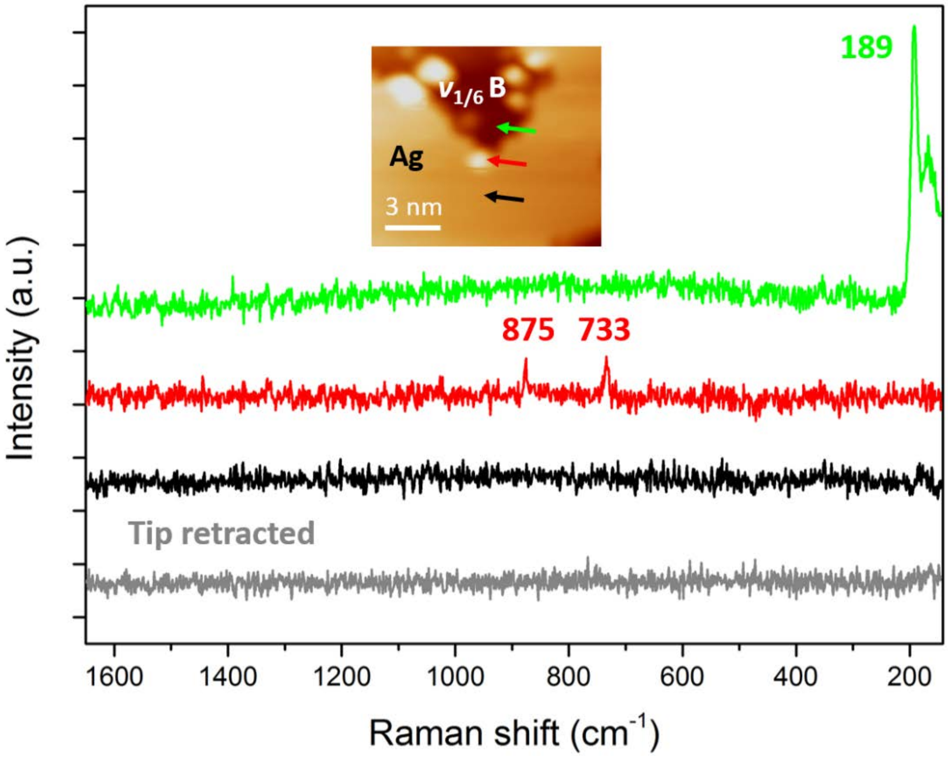

Supplementary Figure 3. TERS measurements on a borophene edge after exposure to atomic oxygen. Green, red, and black spectra were acquired on the $v_{1/6}$ borophene, a small cluster, and the Ag surface, respectively, as marked with arrows. Gray spectrum was collected after tip retraction. TERS measurement parameters: 0.1 V, 3 nA, 5 s. STM scanning conditions: 1.5 V, 300 pA.

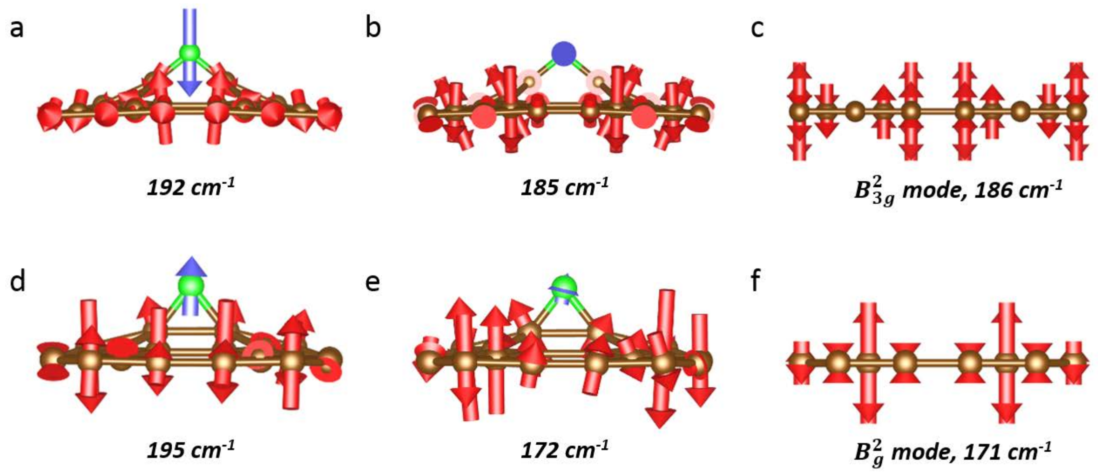

Supplementary Figure 4. Side views of atomic displacements of simulated Raman modes of O-adsorbed and pristine borophene. a–c, O-adsorbed (a,b) and pristine (c) $v_{1/6}$ borophene. **d–f,** O-adsorbed (d,e) and pristine (f) $v_{1/5}$ borophene. Green spheres and blue arrows indicate oxygen atoms and their displacements, respectively. Corresponding calculated phonon frequencies are presented.

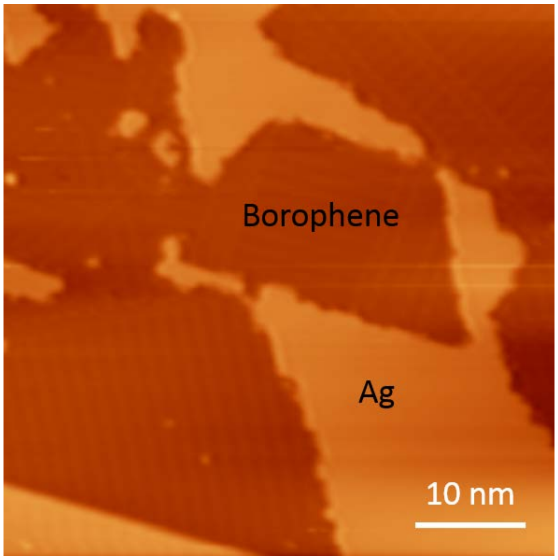

Supplementary Figure 5. STM image of borophene islands after exposure to 6 L of molecular oxygen at room temperature. Scanning conditions: 1.3 V, 150 pA.

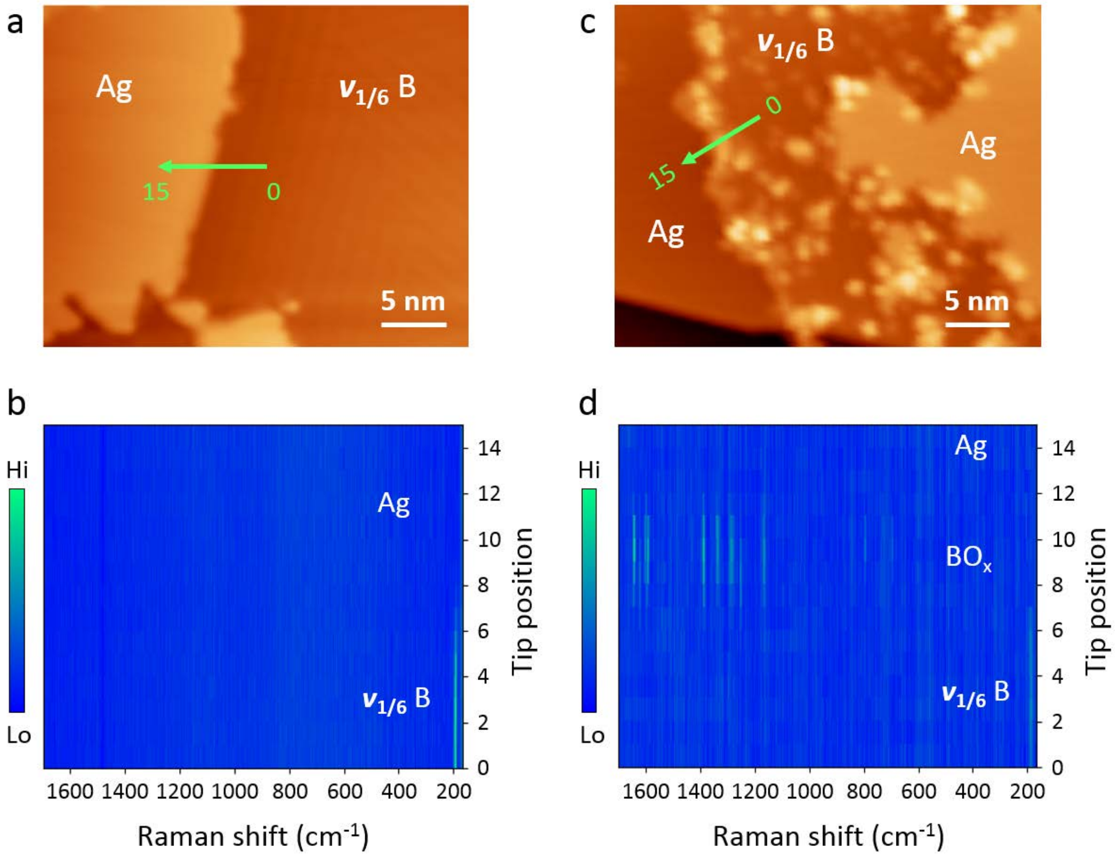

Supplementary Figure 6. Comparison of pristine and oxidized borophene edges. a, STM image of a pristine $v_{1/6}$ borophene edge with a green arrow indicating the tip trace for the TERS line scan shown in (b). **b,** TERS line scan (15 points) along the trace shown in (a). **c,** STM image of an oxidized $v_{1/6}$ borophene edge with a green arrow indicating the tip trace for the TERS line scan shown in (d). **d,** TERS line scan (15 points) along the trace shown in (c). TERS measurement parameters: (b) 0.2 V, 2 nA, 3 s per point, step size ~6.0 Å; (d) 0.2 V, 1 nA, 3 s per point, step size ~4.5 Å. Scanning conditions: (a) 1.3 V, 200 pA; (c) 1.3 V, 100 pA.

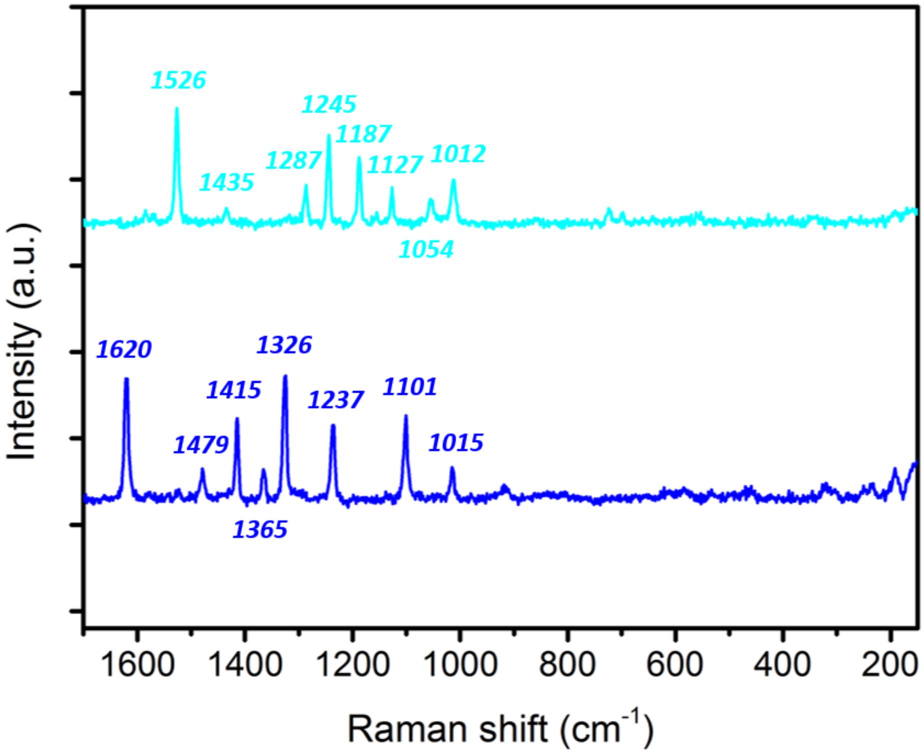

Supplementary Figure 7. Additional TERS spectra acquired at the locations marked with cyan and blue arrows in Fig. 3b. TERS measurement parameters: 0.1 V, 2 nA, 5 s.

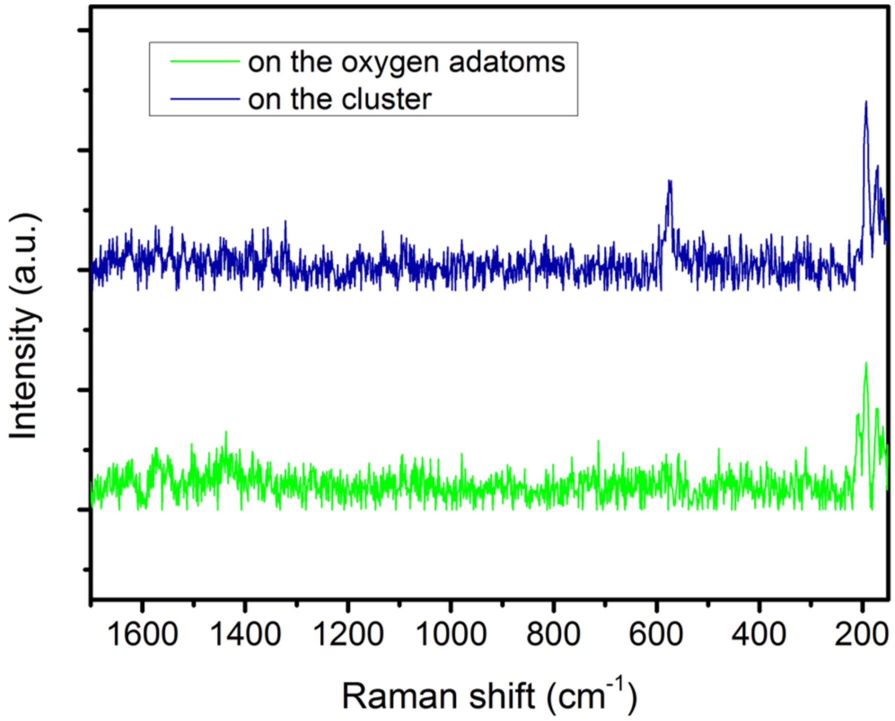

Supplementary Figure 8. Typical TERS spectra acquired when the tip was positioned on top of the high cluster and oxygen adatoms shown in Fig. 3d. TERS measurement parameters: 0.1 V, 3 nA, 2 s.

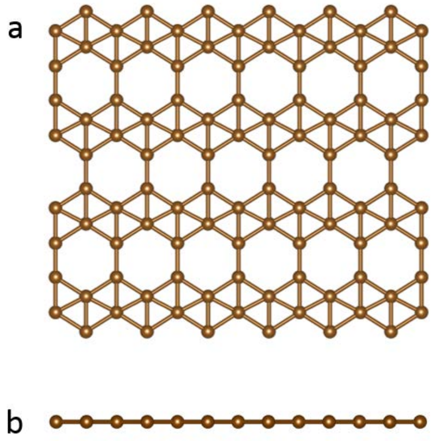

Supplementary Figure 9. Top (a) and side (b) views of the structure of $v_{1/5}$ borophene.

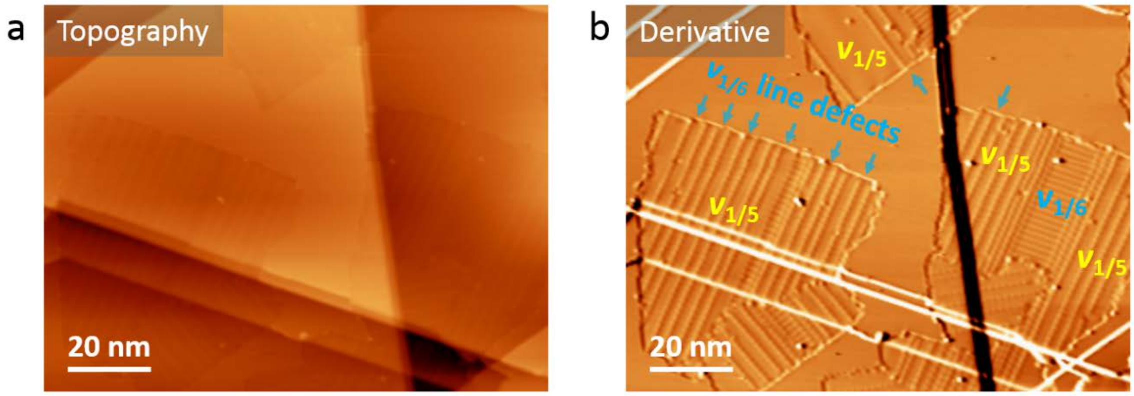

Supplementary Figure 10. Large-scale STM images of borophene line defects. a,b, Topography (a) and the corresponding derivative (b) STM images of borophene islands showing prevalent $v_{1/6}$ line defects in $v_{1/5}$ domains as indicated by blue arrows. Scanning conditions: 2.2 V, 200 pA.

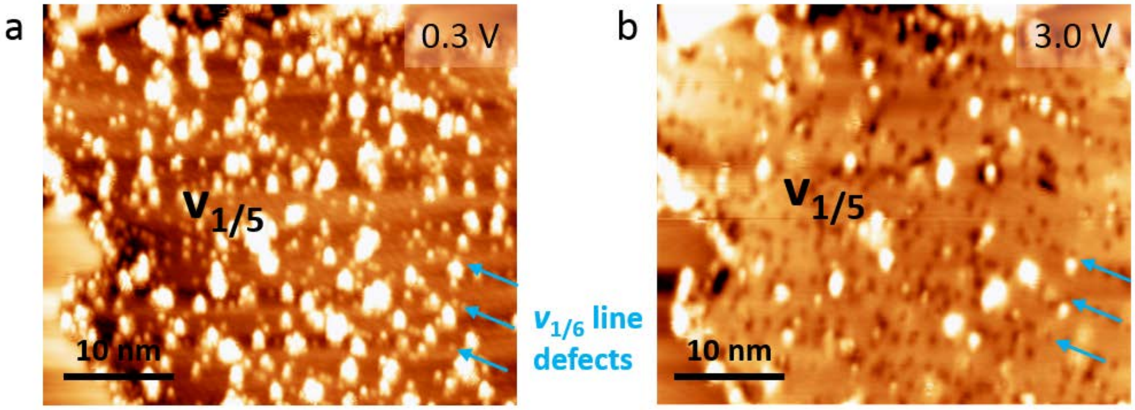

Supplementary Figure 11. Additional STM images of oxidized $v_{1/5}$ borophene. a,b, STM images of a $v_{1/5}$ borophene island following exposure to 4800 L of molecular oxygen, which were acquired at sample biases of 0.3 V (a) and 3.0 V (b), respectively. Oxygen adatoms were imaged as small protrusions (a) and dark dots (b), respectively. Blue arrows indicate the $v_{1/6}$ line defects.

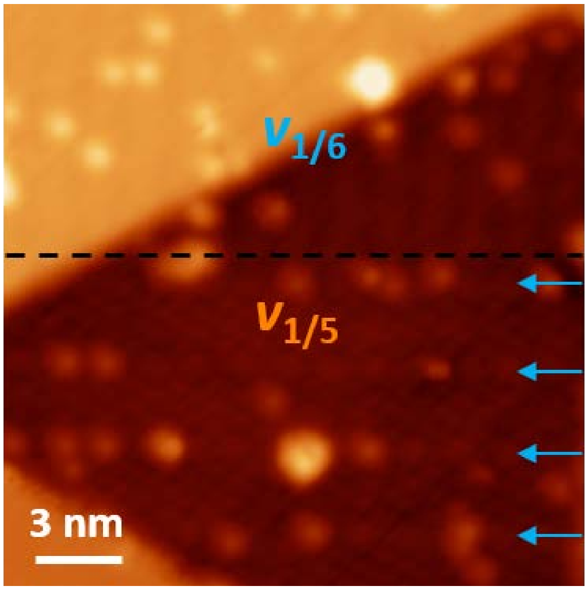

Supplementary Figure 12. STM image of oxidized mixed-phase borophene showing selective oxygen adsorption on the $v_{1/6}$ phase including domains (top part) and line defects (blue arrows). The black dashed line indicates the $v_{1/6} – v_{1/5}$ boundary. Scanning conditions: 1.5 V, 200 pA.

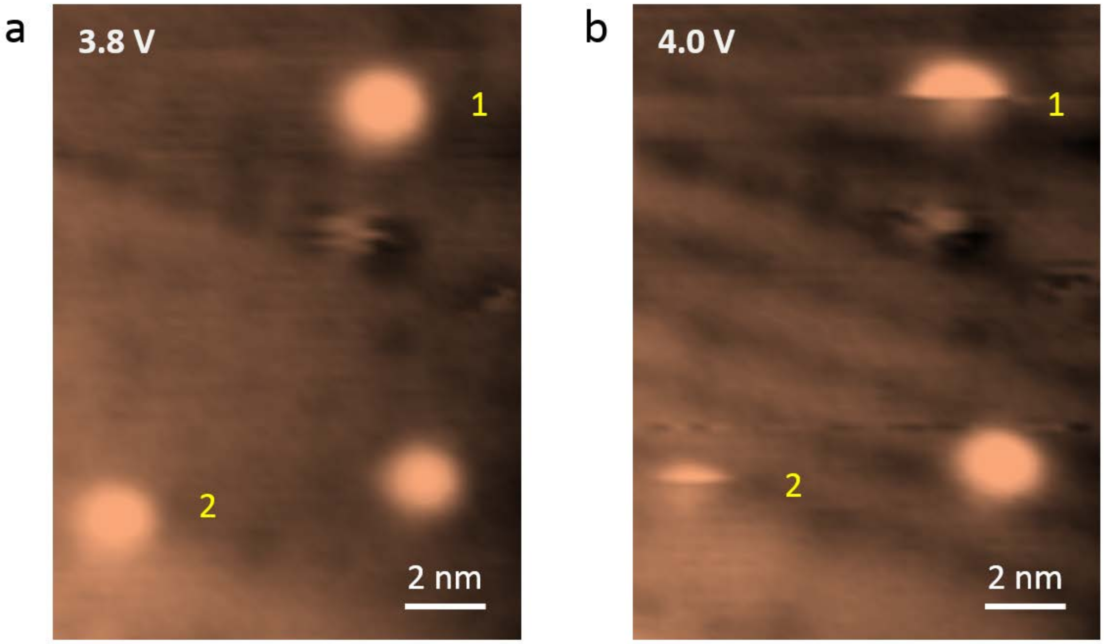

Supplementary Figure 13. Additional STM imaging of the tip-induced decomposition of BO_x clusters. a, STM image before the decomposition showing three BO_x clusters on borophene. Cluster 1 is the same one as shown in Fig. 6b. **b,** STM image recording the bias-induced decomposition of another cluster marked by a 2 in addition to Cluster 1 under the same scanning condition. Scanning conditions: (a) 3.8 V, 150 pA; (b) 4.0 V, 150 pA.

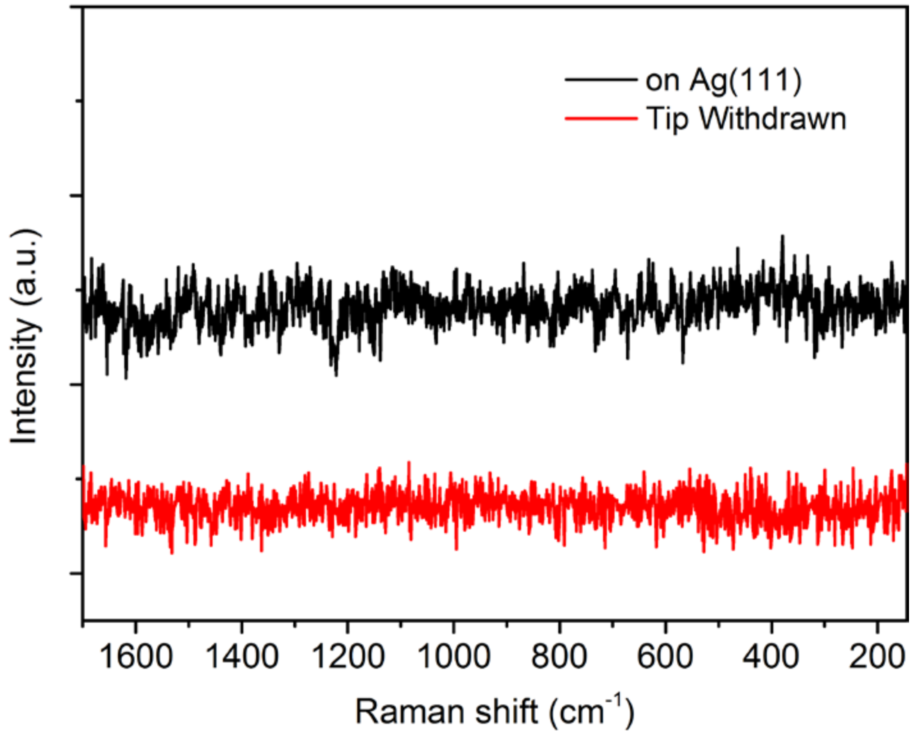

Supplementary Figure 14. TERS spectra acquired on a Ag(111) surface and collected while the tip was retracted. TERS measurement parameters: 0.2 V, 2 nA, 5 s (black); tip withdrawn, 5 s (red).

Supplementary References

1. Mu, Y. W. & Li, S. D. First-Principles Study on the Oxidation of Supported β_{12}-Borophene. *J. Phys. Chem. C* **124**, 28145-28151 (2020).
2. Yang, Q. et al. Crystalline Boron Oxide Nanowires on Silicon Substrate. *Phys. E: Low-Dimens. Syst. Nanostructures* **27**, 319-324 (2005).
3. Liu, X. L., Wang, L. Q., Yakobson, B. I. & Hersam, M. C. Nanoscale Probing of Image-Potential States and Electron Transfer Doping in Borophene Polymorphs. *Nano Lett.* **21**, 1169-1174 (2021).
4. Li, L. et al. Angstrom-Scale Spectroscopic Visualization of Interfacial Interactions in an Organic/Borophene Vertical Heterostructure. *J. Am. Chem. Soc.* **143**, 15624–15634 (2021).
